# Wireless communication system via nanoscale plasmonic antennas

**DOI:** 10.1038/srep31710

**Published:** 2016-08-24

**Authors:** Juan M. Merlo, Nathan T. Nesbitt, Yitzi M. Calm, Aaron H. Rose, Luke D’Imperio, Chaobin Yang, Jeffrey R. Naughton, Michael J. Burns, Krzysztof Kempa, Michael J. Naughton

**Affiliations:** 1 Department of Physics, Boston College, 140 Commonwealth Ave. Chestnut Hill, Massachusetts 02467, USA

## Abstract

Present on-chip optical communication technology uses near-infrared light, but visible wavelengths would allow system miniaturization and higher energy confinement. Towards this end, we report a nanoscale wireless communication system that operates at visible wavelengths via in-plane information transmission. Here, plasmonic antenna radiation mediates a three-step conversion process (surface plasmon → photon → surface plasmon) with in-plane efficiency (plasmon → plasmon) of 38% for antenna separation 4*λ*_0_ (with *λ*_0_ the free-space excitation wavelength). Information transmission is demonstrated at bandwidths in the Hz and MHz ranges. This work opens the possibility of optical conveyance of information using plasmonic antennas for on-chip communication technology.

Recently, in-plane wireless communication systems are being developed to be compatible with the modern on-chip technology^1^. Some of the advantages that in-plane wireless communication offers are, among others, lower losses and reduction in the number of required waveguides^1^ or even electrical isolation^2^. Most modern on-chip optical technology uses near-infrared wavelengths^3^, but for visible wavelengths, an ideal candidate to perform in-plane communication is the surface plasmon (SP), *i.e*. the collective oscillation of electrons coupled to an electromagnetic field at a dielectric-metal interface. As has been demonstrated, SPs have the capability to highly confine energy on the interface where they propagate on subwavelength scales^4^. An additional property of SPs is their capability to be strongly confined to the surface of metallic subwavelength structures, including implementations called plasmonic antennas (PAs)^5^. In this sense, PAs exploit the special properties of the localized surface plasmon. It has been demonstrated that specially designed PAs can collect free-space radiation (photons) and convert it into propagating surface plasmons (SPs) by a momentum up-conversion process (*k*-UC)^6^,^7^,^8^,^9^. Conversely, PAs can perform a momentum down-conversion process (*k*-DC) by converting SPs into photons^10^,^11^. In this sense, several reports have appeared using PAs as receivers or broadcasters of electromagnetic radiation^12^,^13^,^14^. One limitation of these systems is that the free-space radiation is emitted predominantly out-of-plane^15^,^16^, and little effort has been done to facilitate in-plane emission and collection, *i.e.*, in the direction of the SP propagation^17^,^18^. Such an in-plane communication concept could be a significant advancement in on-chip photonic technology, due to better impedance matching between the emitted and received radiation.

Here, we present the realization of the first nanoscale wireless communication system (nWCS) operating at visible wavelengths and based on plasmonic antennas. Such a system is implemented in an in-plane configuration, meaning it allows information transmission and recovery via SPs propagating in the same plane. Communication is achieved across a distance of several wavelengths. We demonstrate the operation of our system by using near-field scanning optical microscopy (NSOM). Numerical calculations confirm the operational principle of the realized system and show good agreement with experimental data. Beyond proof-of-concept, we show two example applications of the system for in-plane information transmission.

## Results

### Numerical optimization

**Figure 1:**
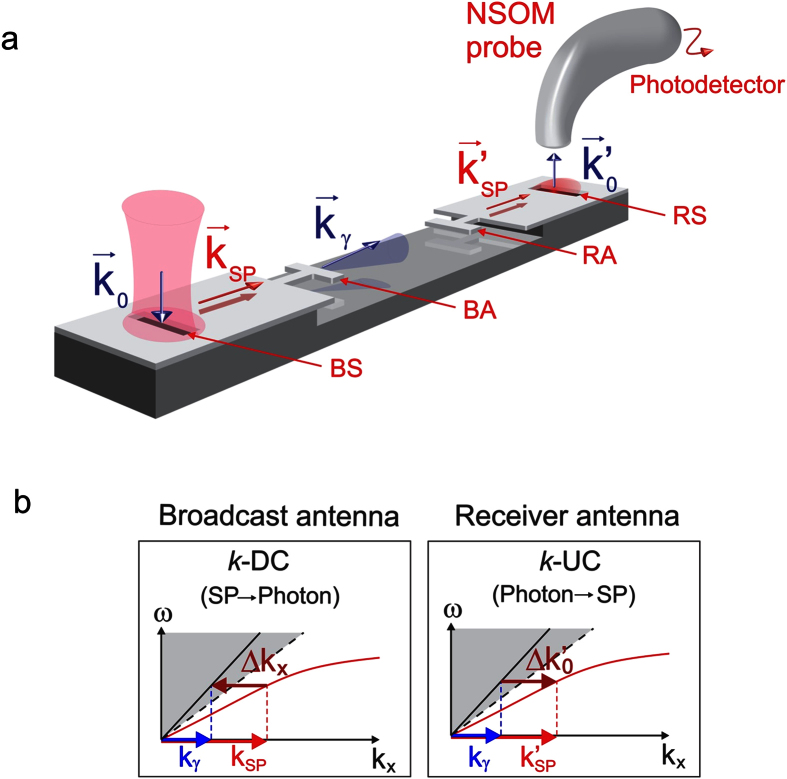
Operation principle of the proposed nWCS scheme. (**a**) Schematic representation of the system, where the arrows represent the momentum of each interacting electromagnetic entity. An NSOM probe for detecting the final photon is depicted. BS and BA refer to broadcast slit and antenna, while RS and RA to receiver slit and antenna, respectively. (**b**) Dispersion relations of the three-step momentum conversion process, where the shaded zones represent the available momentum modes and the dashed lines the light line. The red line represents the SP branch and the magnitudes of Δ*k*_*x*_ and 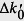 were exaggerated to make clear the *k*-UC and *k*-DC.

The conceptual scheme of the nWCS operation is shown in Fig. 1a. A vertically-incident photon 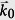 generates a SP via *k*-UC by interacting with the slit in the broadcast region (BS in Fig. 1). The SP, indicated by 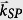, propagates across the broadcast region and decays to photon 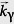 via *k*-DC at the broadcast antenna (BA in Fig. 1). The radiated photon travels across the inter-antenna (free) space to the receiver antenna (RA in Fig. 1), which collects the propagating photon and converts it into SP 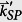 via *k*-UC, which propagates in the receiver region. Finally, this SP decays to photon 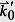 via *k*-DC when it interacts with the slit in the receiver region (RS in Fig. 1). As can be seen, the processes at the slits do not explicitly affect the communication process being considered, but they were included for SP generation (broadcast) and symmetry (receiver) purposes. A simplified version of the nWCS operation is presented as dispersion schemes in Fig. 1b; the dispersion in the inter-antenna space is not included because the photon does not change its nature there.

Our main interest is in optimizing the transfer of propagating SPs from the broadcast to the receiver region by free-space radiation – hence it is not centered on the net emissive power of the PAs, but on the in-plane transmitted power. Thus, we define an efficiency 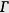 as the ratio between the powers measured in the broadcast and receiver regions, *i.e*., *Γ*=*P*_*receiver*_/*P*_*broadcast*_, understanding the power as the integrated near-field intensity over the measured area.

The nWCS design was optimized by numerical calculation of the maximum efficiency as a function of metallic film thickness (*T*), air-cavity depth below the broadcast and receiver regions (*C*_*d*_), and antenna arm length (*L*_*a*_); see Supplementary Fig. S1 in the Supplementary Information for a definition of nWCS dimensions. For the calculations, we used a three-dimensional numerical model based on the finite element method (COMSOL). The light source was linearly polarized (transverse magnetic) with wavelength 660 nm (454 THz). We used Ag as the plasmonic metal due to its good plasmonic response at the working wavelength and long SP propagation length. The dielectric constants were set as: air = 1.00, glass = 2.25, and Ag = −20.15 + 0.46i. During the optimization process, we defined the inter-antenna distance as 4*λ*_0_ because it is the maximum distance that we explored experimentally.

**Figure 2:**
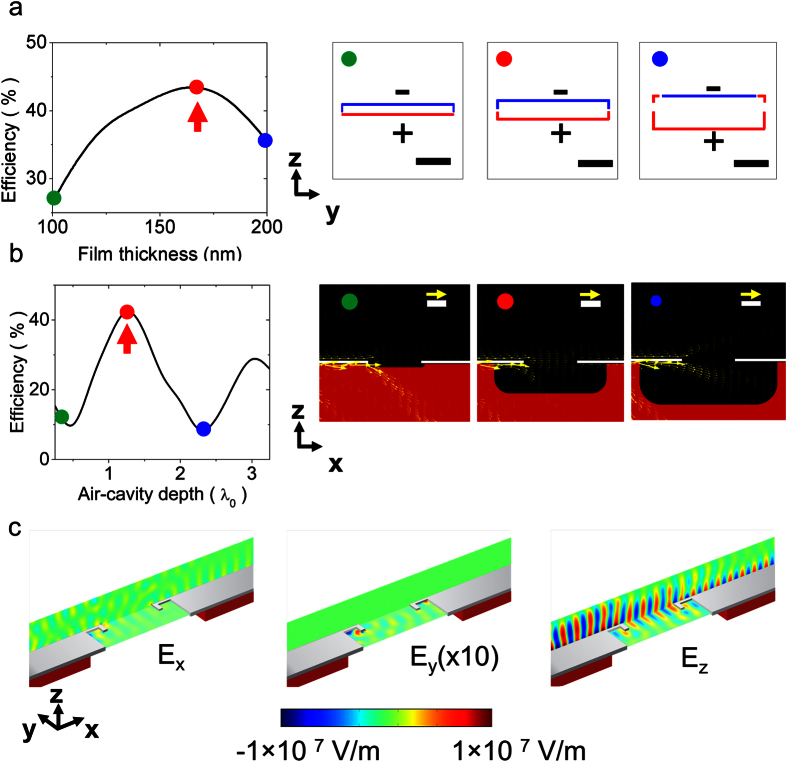
Numerical optimization of the nWCS. (**a**) Efficiency as a function of Ag film thickness. The inset shows the surface charge density calculated at the front face of the broadcast antenna: the film thickness corresponds to the red arrow in the plot; the red and blue outlines correspond to positive and negative charge, respectively, and the scale bar is 500 nm. (**b**) Efficiency as a function of the air-cavity depth below the broadcast and receiver regions, using the optimized Ag film thickness. The inset shows the averaged power flow corresponding to the air-cavity depth indicated by the red arrow in the plot. The scale bar represents 1 μm and the yellow arrow 5 × 10^−5^ W, while the black, white, and red zones represent air, Ag, and glass, respectively. (**c**) Calculated electric field components, with optimized parameters corresponding to the red arrows in (**a,b**). The color bar at the bottom is the same for each of the three field-component images. Note the coordinate references in each image.

As previously stated, the slit in the broadcast region produces the initial *k*-UC, creating SPs whose *E*-field is polarized primarily in the vertical direction. As can be seen in Fig. 2a, the maximum efficiency is obtained when the thickness of the Ag film (*T*) is tuned to generate a dipolar resonant condition at the broadcast antenna edge, see inset. Different modes are excited in the PA edge as the thickness is tuned, but only the dipolar mode allows efficient in-plane information transmission, due to the symmetric radiation generated by the upper and lower edges. In our case, the dipolar condition is obtained when the film thickness is about one fourth the excitation wavelength. This follows from rescaling of the SP wavelength due to the strong confinement at the PA edge^19^. This confinement causes the lateral dimensions (antenna arm lengths) of the PAs to have little effect on the free-space transmission efficiency, affecting only the collimation of the beam (Supplementary Fig. S2).

The air-cavity depth (*C*_*d*_) below the antenna regions plays an important role because the absence of the substrate, which has a higher refractive index than air, is necessary for the photons to propagate close to the horizontal direction (−5° with respect to the horizontal) in the inter-antenna space, as seen in Supplementary Fig. S3. The equivalent case would be a system surrounded by a medium identical to the substrate. We have avoided such inclusion of a high refractive index material because, although it would provide higher confinement of SPs on the metallic surface, it would generate an additional loss channel, reducing the SP propagation length^20^. The shape of the air-cavity below the broadcast and receiver regions was chosen in the simulations to mimic the isotropic etch fabrication process used in the experimental procedure, as will be seen later. Figure 2b shows the calculated efficiency when the air-cavity depth is modified; as expected, the efficiency shows resonant behavior because the finite length of the broadcast region allows the SPs to resonate on the top and bottom surfaces. The effect of the air-cavity depth on the electromagnetic power flow, shown at the inset, demonstrates that only the optimum condition allows the power flow to be horizontal, resulting in an efficient in-plane information transmission, per the image identified with the red circle in the inset to Fig. 2b.

Once the maximum efficiency was obtained, the free-space transmission was characterized by calculating the components of the electric field. In Fig. 2c, the vector components of the electric field, using the optimized parameters, are displayed. The component *E*_*z*_ was found to dominate this transmission, as would be expected considering the vertical polarization of the SP E-field. As stated above, due to the symmetry of the nWCS, the effects of *E*_*x*_ and *E*_*y*_ are negligible for the free-space transmission process and consequently do not affect the information transmission. Finally, a confirmation of the maximum efficiency obtained with the proposed excitation wavelength is shown in Supplementary Fig. S4.

### Sample fabrication

**Figure 3:**
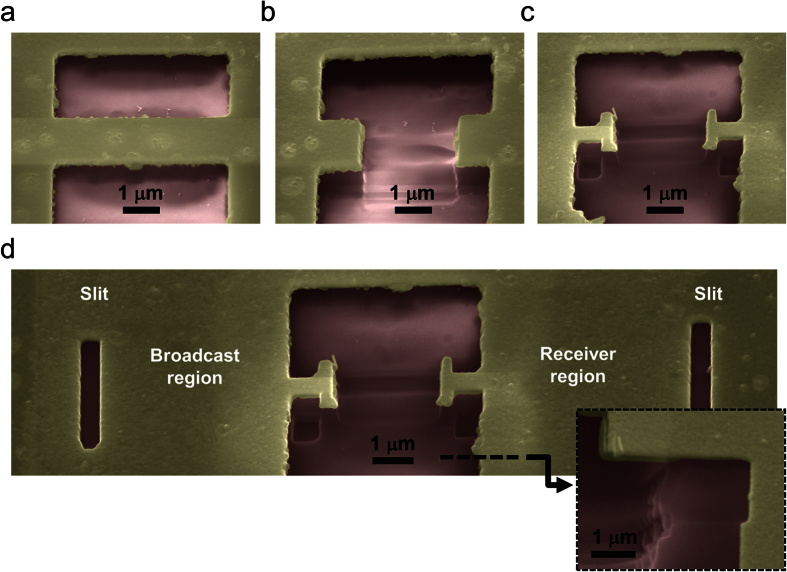
Fabrication process. (**a**) Parallel rectangles milled on the Ag surface after wet etching of glass substrate. (**b**) Fabrication of the inter-antenna space. (**c**) Fabrication of the antenna shapes. (**d**) Final system after fabrication of slits. Inset: Transverse cut realized in the dashed line on **d** showing the shape of the air-cavity below the receiver region. All the cases are false color SEM images where the red and yellow zones represent, respectively, glass and silver; the sample was tilted 45°.

Using the optimized parameters, the nWCS was fabricated by the following procedure: a Ag film of ~170 nm thickness was deposited onto a clean glass substrate by sputtering with a Ti adhesion layer of ~5 nm thickness. Using focused ion beam (FIB), two parallel rectangles were milled on the Ag film. The air-cavity below the broadcast/receiver regions was fabricated by wet-etching the glass substrate by immersion of the sample in buffered oxide etch solution. A second FIB milling created the inter-antenna space, and the broadcast/receiver antennas were fabricated in a third milling process. The nWCS was completed by FIB milling the slits in the broadcast/receiver regions. Samples were fabricated with inter-antenna distance ranging from 0.5*λ*_0_ to 4*λ*_0_ in steps of 0.5*λ*_0_. Figure 3 shows the fabrication process by colored SEM images for an inter-antenna distance of 4*λ*_0_. We did not include the image of the first fabrication process because the similarity to Fig. 3a, where the parallel rectangles were milled as well the substrate was wet-etched. Figure 3b shows the fabricated inter-antenna space, while Fig. 3c shows the milled antennas in the next step. Finally, in Fig. 3d the finished nWCS is displayed, including the slits. A transverse cut was made in the receiver region in order to see the cavity shape below such a region, inset to Fig. 3d. In a second transverse cut in the broadcast region, we confirmed the symmetric shape due to the isotropic etching of the substrate (not shown). Due to the experimental conditions and the fabrication capabilities, the error in the inter-antenna distance was measured as 10% and included later as horizontal error bars in the efficiency measurement report, see below.

### Wireless communication transmission

Due to the localized nature of SPs, a commercial NSOM (Nanonics Multiview 4000) was used to observe the SP behavior at the sample surface. The NSOM probe had a metallic coating (Cr + Au also from Nanonics) and a 300 nm aperture diameter. Scanning was performed in contact mode with a resolution of 25 nm/point and speed of 24 ms/point over an area of 5 × 5 μm^2^. The near-field intensity transmitted by the NSOM probe was measured by using a photon-counter and a signal amplifier. Additionally, the scanning over the sample surface was limited to the planar zones of the broadcast and receiver regions in order to preserve the probe integrity. This is due to the large depth of the bottom glass surface in the inter-antenna space – regardless of the integration time per point in the scanning, the probe would incur some damage, resulting in topographic and intensity artifacts. The light source was a laser diode, linearly polarized, with wavelength of 660 nm and ~50 mW output power, focused to a spot of ~1.5 μm diameter by a microscope objective (50×, NA = 0.5). The polarization of the light beam was set perpendicular to the broadcast slit edge in order to maximize SP generation.

**Figure 4:**
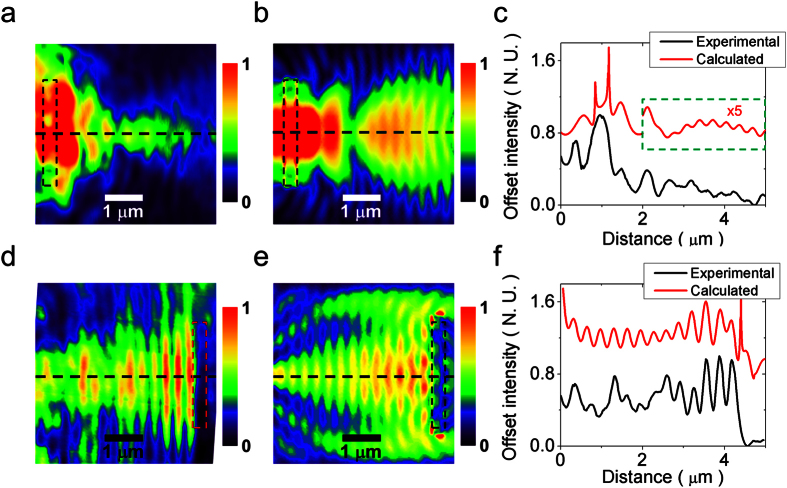
Experimental demonstration of the wireless communication. (**a**) Normalized experimental near-field intensity in the broadcast region. (**b**) Normalized intensity numerically calculated in the broadcast region. (**c**) Intensity transverse cut realized in (**a**,**b**) at the dashed lines. Note the magnification on the dashed rectangle to make clear the intensity features. (**d**) Normalized experimental near-field intensity in the receiver region. (**e**) Normalized intensity numerically calculated in the receiver region. (**f**) Intensity transverse cut realized in (**d**,**e**) at the dashed lines.

Figure 4a shows the experimental near-field intensity in the broadcast region for an inter-antenna distance of 1.5*λ*_0_. From these data, it is clear that the *k*-UC at the broadcast region occurs at the slit and the SPs propagate left-to-right toward the broadcast antenna. Due to the well-matched impedance between SPs and photons at the broadcast antenna, there is a weak interaction between the incoming and the reflected SPs on the metal edge. The numerically calculated near-field intensity is shown in Fig. 4b. In order to make a clear comparison, a transverse cut was made at the dashed line in Fig. 4a,b and the results shown in Fig. 4c as offset intensities. Due to the limited resolution on the NSOM probe, the fine details reported at the slit edge in the calculated data are lost, however, the contrast in the experimental data at the slit edge is lower and the features in the near-field intensity are better defined.

Next, Fig. 4d shows the experimental near-field intensity in the receiver region. Such intensity is modulated by a pattern that is evidently a SP standing wave. This standing wave is the result of interference between the SPs converted from photons (*k*-UC) at the receiver antenna and the SPs reflected from the receiver slit; the pitch of the pattern agrees well with the SP wavelength (Supplementary Fig. S5). By comparing the last result with that numerically obtained (Fig. 4e), it is clear that the calculated result is consistent with the experimental results. The comparison was made by a transverse cut realized at the dashed line in Fig. 4d,e and shown in Fig. 4f. Although there is good coincidence between the profiles, as before, the NSOM probe resolution produced a loss of information at the slit edges.

**Figure 5:**
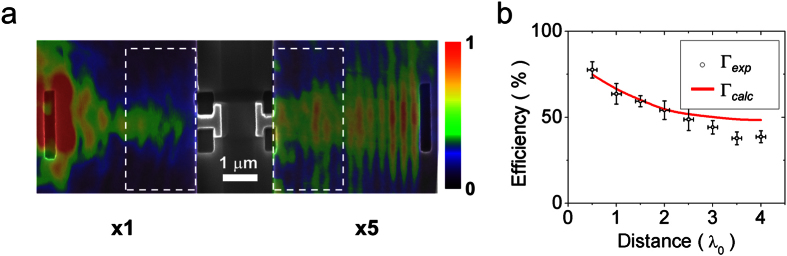
Near-field observation of the wireless transmission process and measured efficiency. (**a**) Normalized experimental near-field intensity superimposed onto SEM image on the nWCS. The receiver region intensity has been multiplied by 5 for comparison purposes. (**b**) Comparison between calculated (*Γ*_*calc*_, red line) and experimental (*Γ*_*exp*_, black circles) efficiencies. The vertical error bars represent the standard deviation of the efficiency measured after three experiments. The horizontal error bars represent the inter-antenna distance error.

The experimental near-field intensity on the broadcast/receiver surfaces (Fig. 4a,d) is displayed as a color profile superimposed on an SEM image, Fig. 5a. There, the near-field intensity in the receiver region was enhanced in order to be clearly distinguished on the same scale as the intensity reported at the broadcast region. As expected, in the receiver region, the SP coupling is done mainly in the PA, proof of this is the well localized beam behind the PA position. The complete set of intensities measured in the receiver region is shown in Supplementary Fig. S6. In the case of no slit in the receiver region, as expected, the SPs will propagate without reflection and consequently there will be no SP standing wave; the near-field image in such a case is presented in Supplementary Fig. S7.

Following our efficiency definition, the power was measured experimentally and calculated numerically as the integrated intensity over an area of 2 × 5 μm^2^ behind the broadcast/emitter regions edges, see dashed rectangles in Fig. 5a. These areas where chosen in order to measure only the near-field components of the signal, *i.e*. avoid any propagative component from the light source and metal edges. The experimental (*Γ*_*exp*_) and calculated (*Γ*_*calc*_) efficiencies are shown in Fig. 5b; the calculated efficiency is quite consistent with the experimental results. A difference in efficiencies is clear in the cases where the inter-antenna distance is longer than ~2*λ*_0_. This difference is due to power losses from the Ag surface roughness and the mismatched coupling produced by the plasmonic antennas defects. The former implies that the nWCS efficiency, for distances of several wavelengths, can be improved by using atomically smooth surfaces^21^,^22^. Additionally, our experimental result follows typical inverse-square power density decay (~1/*r*^2^) of antenna radiation in the far-field region^23^, *i.e*. at distances longer than 2*λ*_0_ as shown in Fig. S8. In Fig. 5b, the vertical error bars were calculated as the standard deviation of the obtained efficiencies after a repetition (three times) of the experiment, while the horizontal bars as the fabrication defect in the inter-antennas distance, as stated before.

The last results are the main achievement of the present work, demonstrating that the nWCS allows the information transmission beyond the near-field interaction distance, *i.e*., along a distance of several wavelengths. From radio frequency theory, the far-field region of an antenna is defined as any distance beyond 2*D*^2^/*λ*_0_, with *D* the maximum linear dimension of the antenna^23^. In our case, the far-field region is located beyond 2*λ*_0_. This means we have indeed coupled plasmonic antennas in the far-field on an in-plane scheme. To our knowledge, this is the first time this phenomenon is reported and applied for information transmission.

### Wireless communication demonstration

**Figure 6:**
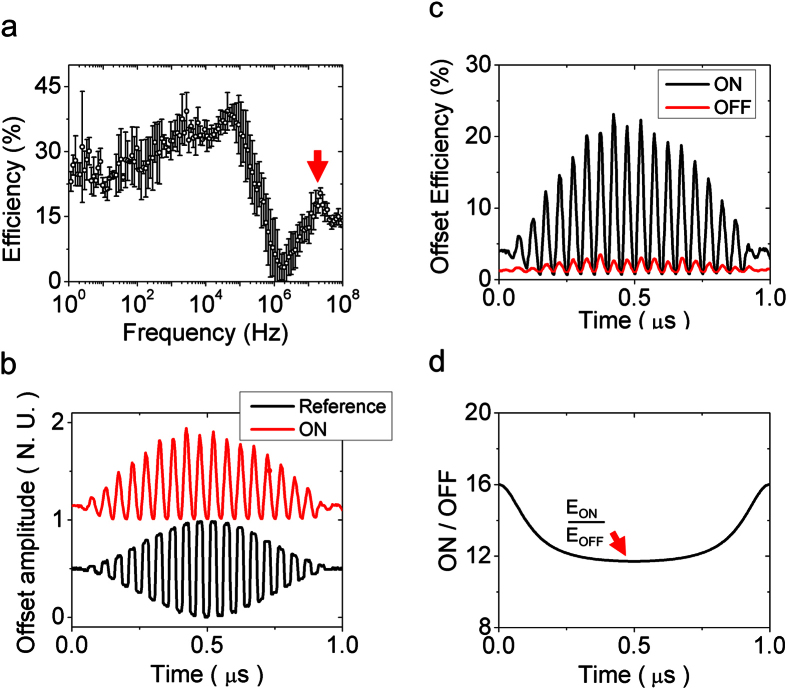
Information transmission demonstration. (**a**) Efficiency of the system as a function of the driving frequency. The red arrow shows the used frequency. (**b**) Comparison between the reference (black line) and the detected (red line, offset for clarity) signals, normalized to their maximum values. (**c**) Comparison between the efficiencies on the ON and OFF slit states. (**d**) Ratio between the envelope amplitudes of signals corresponding to ON and OFF slit states measured at the maximum efficiency (red arrow).

While the objective of this work is proof-of-principle of in-plane wireless communication using PAs at optical frequencies, we also performed data transmission experiments in the near-infrared to show one possible application of the nWCS for in-plane communication. In all the cases, the inter-antenna distance was set to 2*λ*_0_. The light source was a laser diode, linearly polarized, with wavelength of 780 nm and ~100 mW output power and focused to a spot of ~2.5 μm diameter. The laser intensity was modulated with a function generator by using a square signal (5 Vpp and offset of 2.5 V). Detection was done using the NSOM described above using a probe with an aperture diameter of 2 μm to enhance the detected intensity. The probe was set to contact mode in a fixed position at the center of the receiver region. The photon-counter signal was measured on an oscilloscope and averaged over 2,048 samples. The frequency was swept from 1 Hz to 100 MHz in 122 points and the efficiency was measured at each frequency. The information transmission efficiency as a function of the driving frequency of the complete system (light source, nWCS, NSOM probe and photodetector) is shown in Fig. 6a. The higher efficiency shown at the kHz range is due to the laser diode source performance and is not related to the nWCS, because the maximum information transmission speed of the plasmonic system is limited only by the dispersion of the metal used. Using a 20 MHz signal, where a local maximum was obtained in the frequency response (red arrow in Fig. 6a), and modulated by an envelope sinusoidal signal at 1 MHz, it is clear that the system reproduces finely the reference signal, Fig. 6b.

We found that due to the Ag surface roughness, the generation of SPs is inevitable when the light beam is illuminating a region different from the slits, *i.e*. a *flat* region. We have defined the ON slit and OFF slit states as the states where the beam is impinging the broadcast slit and a *flat* surface respectively, see Supplementary Fig. S9. In Fig. 6c, we show both the ON and OFF slit state signals; indeed, it was found that the ON/OFF slit state signal ratio is of the order of 12, as shown in Fig. 6d. This ratio is enough to clearly distinguish between the state signals, and can be strongly increased by reducing the surface roughness of the metal film.

An additional demonstration of information transmission by the nWCS was done using frequencies in the audible range (Hz to kHz) and reported in the Supplementary Video. A piezoelectric buzzer was used to transduce the electrical signal generated in the signal amplifier into an audible sound. The signal was modulated by a square wave (5 Vpp and offset of 2.5 V) with a burst of 5 cycles at 40 Hz and trigger interval of 500 ms. The buzzer tone (centered at 3.6 kHz), is heard in the Supplementary Video. It is demonstrated that the position change of the NSOM probe as well as the broadcast slit with respect to the light source determines the signal strength, as expected by the high sensitivity of SPs to the metallic surface characteristics.

## Discussion

In summary, we have experimentally demonstrated the first approach to high efficiency in-plane information transmission using SP → photon → SP interactions by momentum up- and down-conversion processes. The reported fabrication is a three-step process (FIB-wet etch-FIB), realizing the design in an easy-to-develop architecture. Although the experimental efficiency is of the same order as that numerically calculated, it could be improved by using higher directivity antennas as well as smoother metal surfaces. An application of information transmission was shown, where frequency modulation was chosen for demonstration purposes but the maximum speed of the proposed system is limited only by the dispersion of the plasmonic metal used. Our results could pave the way to a nanoscale and visible frequency equivalent of existing wireless communication systems^24^. Some additional applications of the reported scheme are circuit switching by beam steering, high-efficiency coupling to plasmonic waveguides, and high-speed communication^9^,^25^,^26^. Finally, the proposed in-plane wireless communication process, because some part of such a process is held in free-space, can improve the speed of the information transmission as much as 60% with respect to dielectric-loaded plasmonic waveguides^27^ and 50% to single plasmonic nanowire waveguides^28^, yielding an asset to available technology based on plasmonic systems.

## Additional Information

**How to cite this article**: Merlo, J. M. *et al*. Wireless communication system via nanoscale plasmonic antennas. *Sci. Rep*. **6**, 31710; doi: 10.1038/srep31710 (2016).

## Supplementary Material

Supplementary Information

Supplementary Video S1
