# Supplementary material for: Wireless communication system via nanoscale plasmonic antennas
Source: Sci Rep. 2016 Aug 24;6:31710. doi: 10.1038/srep31710 (PMC4995427; doi:10.1038/srep31710)
Supplement: Supplementary Information [file srep31710-s1.pdf]

SUPPLEMENTARY INFORMATION

Wireless communication system via nanoscale plasmonic antennas

Juan M. Merlo*, Nathan T. Nesbitt, Yitzi M. Calm, Aaron H. Rose, Luke D'Imperio, Chaobin Yang, Jeffrey R. Naughton, Michael J. Burns, Krzysztof Kempa and Michael J. Naughton*

Optimization process

Parameters definition. The optimization process was done by a parametric study on the nWCS dimensions. Such dimensions are defined in Figure S1a, where L_a is the antenna arm length, D_a the extension of the antenna, W_a the width of the free standing elements, T the film thickness, C_d air-cavity depth below the emitter/receiver regions and W_s the slit width; Figure2b depicts the inter-antenna distance D, defined as the space separating the end edges on the plasmonic antennas.

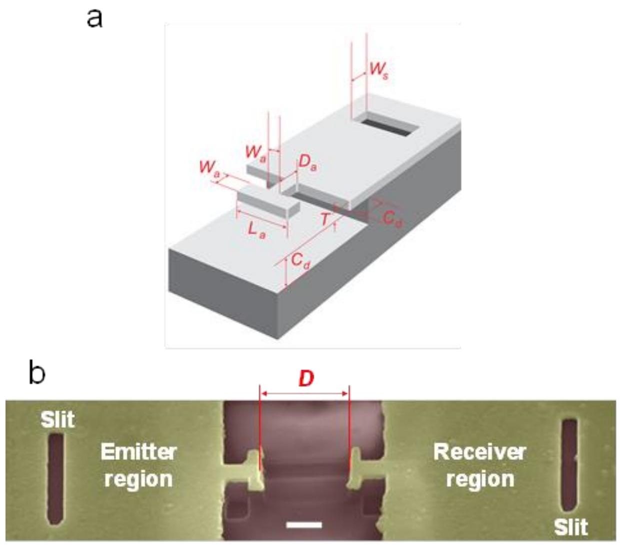

Figure S1. (a) Dimension definitions on the emitter/receiver antenna. (b) inter-antenna distance definition.

Antenna arm length. Due to the distribution charge in the top and bottom surfaces of the broadcast region, the lateral dimensions of the antennas have a small effect in the information transmission. Figure 2S show the efficiency as a function of the antenna arm length (L_a).

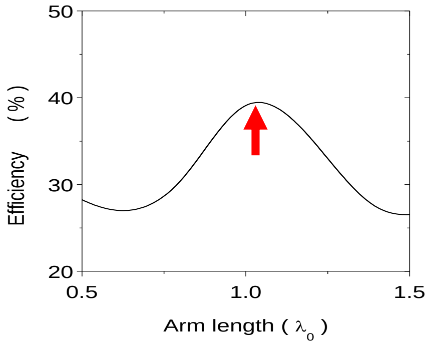

Figure S2. Calculated efficiency dependence on arm length L_a.

Far-field radiation pattern. In order to determine the main direction of the radiation produced by the nWCS, it was calculated the far-field radiation pattern. Figure S3a shows the *E*-plane component of the far-field radiation pattern when the substrates used are air (black line) and glass (red line). Figure S3b shows the *H*-plane component of the far-field radiation pattern following the same conditions as shown in a.

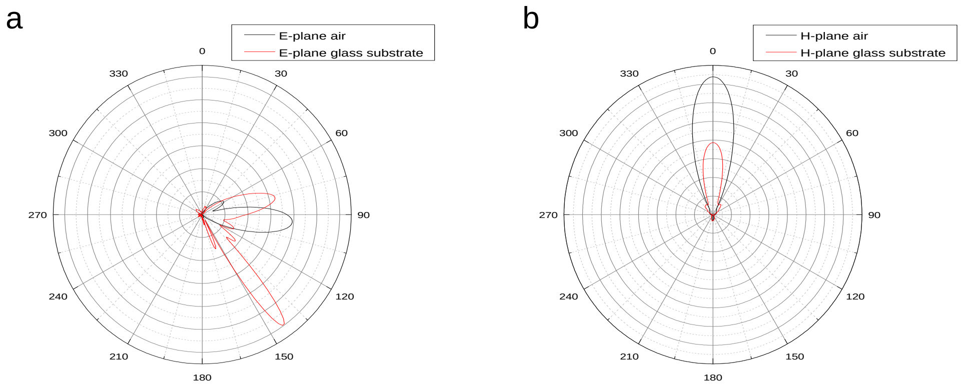

Figure S3. Calculated far-field radiation pattern generated by an nWCS for the E-plane (a) and H-plane (b).

Excitation wavelength. Once the optimized parameters were obtained, it was studied the efficiency as function of the wavelength operation. Figure S4 shows the dependence of calculated efficiency wherethe red arrow represents the excitation wavelength used. The periodic behavior is due to the finite size of the broadcast/receiver regions where the SPs can resonate.

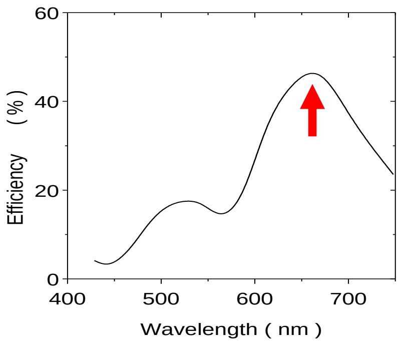

Figure S4. Dependence of calculated efficiency on the operation wavelength.

Experimental results

Surface plasmon wavelength and propagation length. The interaction between the incoming and reflected SPs on the receiver region produces a standing wave that is observed in the near-field intensity. The particular case of an inter-antenna distance of $D = 1.5\lambda_0$ is shown in Figure S4a. The color represents a linear intensity scale with red and black showing the maximum and minimum respectively, while scale bar is 1 μm. An intensity transverse cut was made in the dashed line on Figure S4a is shown in Figure S4b in order to calculate the power spectrum of the resulting intensity profile (Figure S4c). It is clear that the main peak of the power spectrum agrees correctly with half of the SP wavelength as shown by the red arrow, *i.e.* 320 nm. By

measuring the SPP propagation on the sample surface, it was found that the propagation length was 4.4 μm. This value is certainly much shorter than the theoretical one (~80 μm) due to the high roughness and consequently to the strong radiation losses. In this case, all the data manipulation was done in WSxM[1].

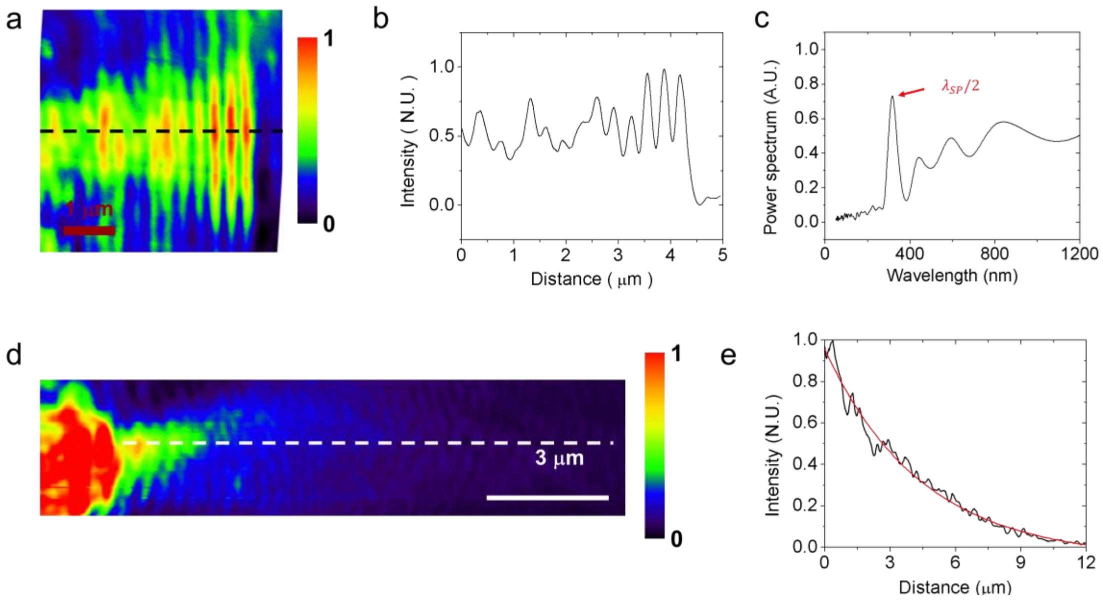

Figure S5. Plasmonic standing wave characteristics measured by NSOM. (a) Near-field intensity. (b) Intensity profile made in the dashed line in (a). (c) Power spectrum of the intensity profile shown in (b). (d) Near-field intensity of a non-confined SP. (e) Black line, intensity profile realized in the dashed line of (d) and, red line, numerical fit.

Near-field intensity at different distances. The normalized near-field intensity registered in the complete set of samples is shown in Figure S6a, where the inter-antenna distance is shown in each image at the top-left. In all the cases, the displayed area is 5x5 μm^2 In order to make an easier comparison between the different images the intensity scale has been adjusted in a factor shown at the top-right of each image. As it is well-known the NSOM is capable to imaging the

near-field intensity at the same time than the topography of the sample. Taking advantage of this, it is possible to localize the position of the interactions held in the sample surface. Figure S6b shows a three-dimensional representation of the sample surface topography with the color corresponding to the near-field intensity measured simultaneously when $D = 1.5\lambda_0$ (red frame in S6a). It is clear that the SP is reflecting on the receiver slit. The data manipulation was done using WSxM[1].

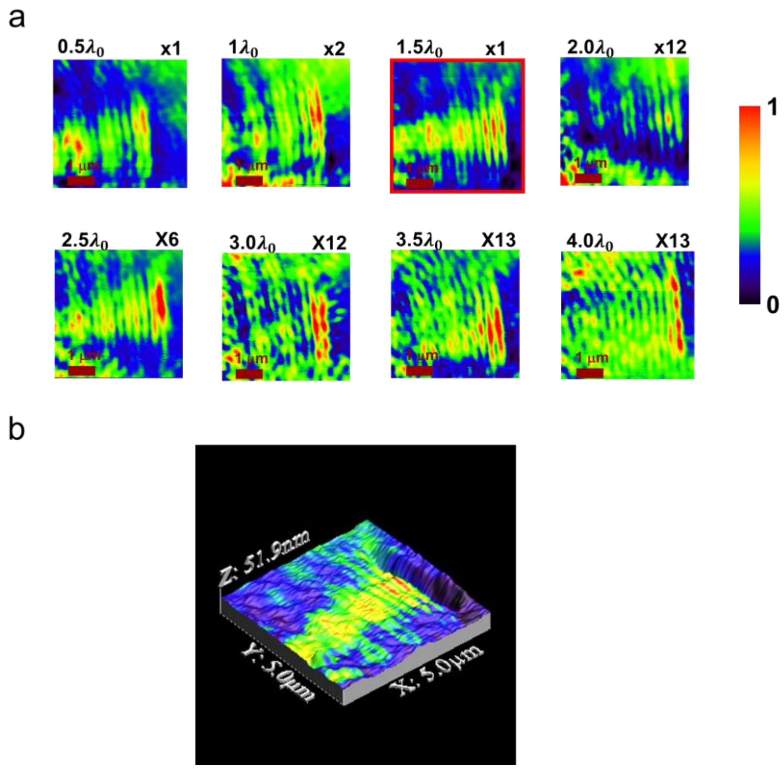

Figure S6. (a) Complete set of near-field intensities for the different inter-antenna distances. (b) Three dimensional representation of the sample topography.

Surface plasmon propagation in the absence of receiver slit. An expected result is that when the receiver slit is not present, the surface plasmons will propagate with no perturbation, meaning the interference pattern shown in Figure S5 is not visible. Figures S7a and S7b show the surface

topography of the receiver region and normalized near-field intensity, respectively, in the case of an inter-antenna distance of $4\lambda_0$. A three-dimensional representation of the sample surface topography with the color corresponding to the near-field intensity is shown in Figure S7c. It is clear the high intensity in the antenna junction to the flat surface (red arrow) due to strong far-field scattering. The data manipulation was done using WSxM[1.]

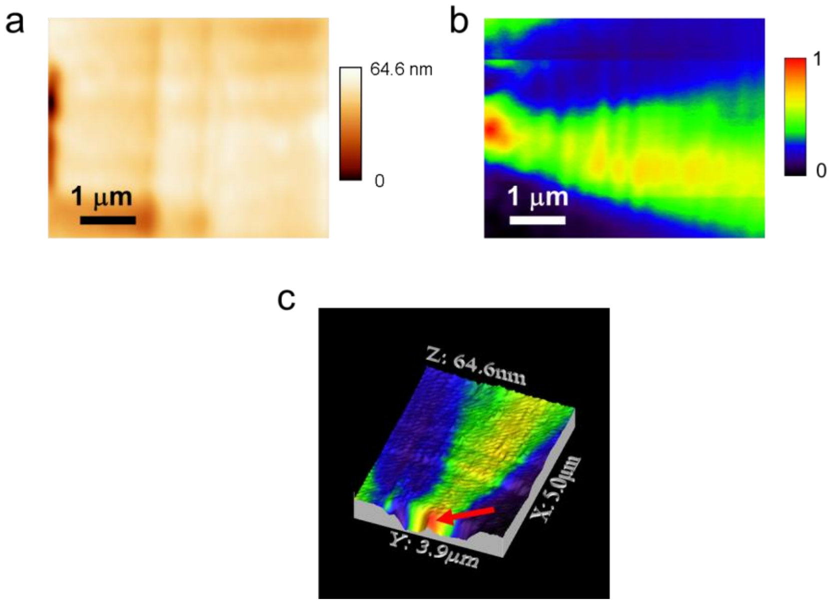

Figure S7. Near-field data in the case where the slit in the receiver region is absent. (a) Sample topography. (b) Near-field intensity. (c) Three dimensional representation of the sample topography.

Information transmission efficiency. The circles represent the experimentally-determined efficiency, while the red line is the numerically calculated. The far-field power density generated by an antenna follows a $1/D^2$ decay[2]. In the particular case of the fabricated structures, this condition is obtained beyond a distance of $\sim 2\lambda_0$; see line blue. It can be seen that the experimental data follow finely the $1/D^2$ relation.

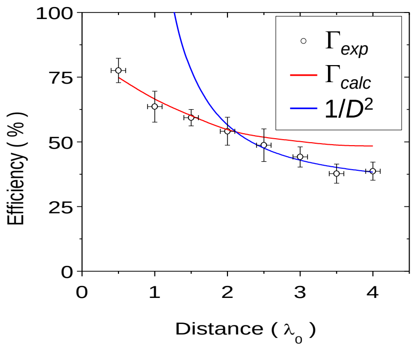

Figure S8. Far-field fitting of the experimental results.

ON/OFF slit states. Due to the finite roughness on the Ag surface, the generation of surface plasmons is inevitable when the light source is illuminating a *flat* region. In such a case, two states have been defined: ON slit state, when the light beam is impinging on the emitter slit, and OFF slit state, when the light is impinging on any *flat* area on the emitter region. Clearly, in the case of a perfectly flat surface, the OFF state must produce a null signal.

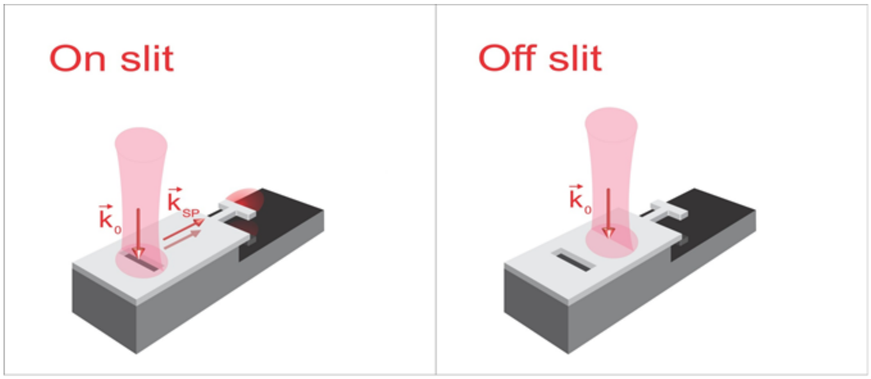

Figure S9. Definition of ON/OFF slit states.

Supplementary Video. In the images associated with this video, the symmetry of the system was mirrored in the horizontal direction with respect to that shown in the main text of this work. A brief description of the Supplementary Video is made as follows:

0:00 – 0:22. A schematic description of the wireless communication process is shown.

0:23 – 0:27. SEM image of the real sample is shown. The inter-antenna distance is $2\lambda_0$.

0:28 – 0:34. A localization of system elements and sample in an optical image is done.

0:39 – 1:00. The displacement of the NSOM probe is done, first by separating and second by approaching to the receiver antenna. In the first case, the sound intensity decreases as a consequence of the SPs decaying intensity. The opposite happens when the probe approaches to the antenna.

1:01 – 1:17. The displacement of the sample is done. The NSOM probe position is fixed with respect to the sample. It can be clearly distinguished four steps when the broadcast slit is on the neighborhood of the light source: first (1:02), the sound is clear and loud when the slit is at the center of the light source (LS); second (1:04), the sound disappear when the slit is moved out of the center of the LS to the first minimum of the Airy disk produced by light source; third (1:06), the sound is clear and weak when the slit reaches the first maximum of the Airy disk produced by the light source; fourth (after 1:08), the sound disappears when the light source is impinging out of the slit. Here, we can distinguish the ON slit state in any time before 1:02, and the OFF slit state can be recognized after 1:08.

References

(1) Horcas, I. et al. *Rev. Sci. Instrum.* **78**, 013705 (2007).

(2) Pozar, D. *Microwave engineering*, Ch. 14, 658 – 674 (John Wiley & Sons, 2012).
